# RET inhibition in novel patient-derived models of RET fusion- positive lung adenocarcinoma reveals a role for MYC upregulation

**DOI:** 10.1242/dmm.047779

**Published:** 2021-02-11

**Authors:** Takuo Hayashi, Igor Odintsov, Roger S. Smith, Kota Ishizawa, Allan J. W. Liu, Lukas Delasos, Christopher Kurzatkowski, Huichun Tai, Eric Gladstone, Morana Vojnic, Shinji Kohsaka, Ken Suzawa, Zebing Liu, Siddharth Kunte, Marissa S. Mattar, Inna Khodos, Monika A. Davare, Alexander Drilon, Emily Cheng, Elisa de Stanchina, Marc Ladanyi, Romel Somwar

**Affiliations:** 1 Department of Pathology, Memorial Sloan Kettering Cancer Center, New York, NY 10065, USA; 2 Human Oncology and Pathogenesis Program, Memorial Sloan Kettering Cancer Center, New York, NY 10065, USA; 3 Thoracic Oncology Service, Division of Solid Tumor Oncology, Department of Medicine, Memorial Sloan Kettering Cancer Center, New York, NY 10065, USA; 4 Faculty of Medicine, The Chinese University of Hong Kong, Shatin, NT, Hong Kong SAR, China; 5 Antitumor Core Facility, Molecular Pharmacology Program, Memorial Sloan Kettering Cancer Center, New York, NY 10065, USA; 6 Department of Pediatrics, Oregon Health Sciences University, Portland, OR 97239, USA

**Keywords:** *RET* fusion PDX, MYC, RET inhibitor, Transcriptome profiling, NSCLC

## Abstract

Multi-kinase RET inhibitors, such as cabozantinib and RXDX-105, are active in lung cancer patients with *RET* fusions; however, the overall response rates to these two drugs are unsatisfactory compared to other targeted therapy paradigms. Moreover, these inhibitors may have different efficacies against *RET* rearrangements depending on the upstream fusion partner. A comprehensive preclinical analysis of the efficacy of RET inhibitors is lacking due to a paucity of disease models harboring *RET* rearrangements. Here, we generated two new patient-derived xenograft (PDX) models, one new patient-derived cell line, one PDX-derived cell line, and several isogenic cell lines with RET fusions. Using these models, we re-examined the efficacy and mechanism of action of cabozantinib and found that this RET inhibitor was effective at blocking growth of cell lines, activating caspase 3/7 and inhibiting activation of ERK and AKT. Cabozantinib treatment of mice bearing RET fusion-positive cell line xenografts and two PDXs significantly reduced tumor proliferation without adverse toxicity. Moreover, cabozantinib was effective at reducing growth of a lung cancer PDX that was not responsive to RXDX-105. Transcriptomic analysis of lung tumors and cell lines with RET alterations showed activation of a MYC signature and this was suppressed by treatment of cell lines with cabozantinib. MYC protein levels were rapidly depleted following cabozantinib treatment. Taken together, our results demonstrate that cabozantinib is an effective agent in preclinical models harboring *RET* rearrangements with three different 5′ fusion partners (*CCDC6*, *KIF5B* and *TRIM33*). Notably, we identify MYC as a protein that is upregulated by RET expression and downregulated by treatment with cabozantinib, opening up potentially new therapeutic avenues for the combinatorial targetin of RET fusion- driven lung cancers. The novel RET fusion-dependent preclinical models described here represent valuable tools for further refinement of current therapies and the evaluation of novel therapeutic strategies.

## Abstract (precis)

**Summary:** Establishment of four patient-derived models of RET fusion-positive lung adenocarcinomas with three different RET fusions shows that MYC expression is regulated by RET.

## INTRODUCTION

The rearranged during transfection (*RET)* gene encodes a proto-oncogene and was identified in 1985 (Takahashi et al., 1985). The gene maps to chromosome 10q11.2 and encodes a single-pass transmembrane receptor tyrosine kinase consisting of three domains: an extracellular domain, a transmembrane domain and a tyrosine kinase domain. The primary RET ligands belong to the glial-derived neurotrophic factor (GDNF) family, which activates RET by first binding to their cognate receptor (GDNF receptor alpha) and then the GDNF-GRFα complex dimerizes with RET, leading to its autophosphorylation and activation. The phosphorylated (phospho)-tyrosine residues on RET serve as docking sites for the SH2 domains of several signaling molecules that activate downstream pathways (e.g. RAS/MAPK, PI3K/AKT) associated with cellular proliferation, migration, and differentiation (Arighi et al., 2005; Phay and Shah, 2010).

Rearrangement of the RET protein through inter- or intra-chromosomal translocation, in which the kinase domain of *RET* is fused with another gene, results in a fusion oncogene (Stransky et al., 2014). Fusion of the kinase domain of *RET* to the 5′ untranslated region (5′-UTR) of *CCDC6* was initially discovered in papillary thyroid carcinoma (Fusco et al., 1987). Subsequently, fusion of *RET* with various other upstream partners were discovered in lung (Li et al., 2017; Lipson et al., 2012; Suehara et al., 2012; Takeuchi et al., 2012) and other cancers (Le Rolle et al., 2015; Paratala et al., 2018). In non-small cell lung carcinoma (NSCLC), *RET* fusion with 5′-UTRs have been described for at least twelve partner genes, i.e. *KIF5B*, *CCDC6*, *NCOA4*, *MYO5C*, *EPFA5*, *TRIM33*, *CLIP1*, *ERC1*, *PICALM*, *FRMD4A*, *RUFY2* and *TRIM24* (Ferrara et al., 2018). The N-terminal partner of RET in all fusion variants described to date contributes to a protein-protein interaction (i.e. coiled-coil) domain that aids dimerization of the fusion oncoprotein, allowing for constitutive RET activation by autophosphorylation (Ju et al., 2012). The tumorigenic potential of RET fusion proteins has been demonstrated *in vitro* and *in vivo* (Li et al., 2017; Takeuchi et al., 2012). In addition to gene rearrangements, RET can also be activated by mutations within the kinase domain in medullary thyroid carcinoma (Jhiang, 2000).

Oncogenic RET is an actionable therapeutic target that is found in 1-2% of NSCLCs and 9% of thyroid cancers (Elisei et al., 2013; Zehir et al., 2017). In NSCLCs, several small-molecule RET inhibitors, such as the multi-kinase inhibitors (MKIs) cabozantinib (Drilon et al., 2016), sunitinib (Gautschi et al., 2017), lenvatinib (Gautschi et al., 2017), vandetanib (Yoh et al., 2017), RXDX-105 (Drilon et al., 2019a), or selective inhibitors, such as selpercatinib (LOXO-292) (Drilon et al., 2019b, 2020) and pralsetinib (BLU-667) (Gainor et al., 2019), have been tested in individual patients or clinical trials.

However, the activity of MKIs, including cabozantinib, RXDX-105, vandetanib and sunitinib, in patients with *RET*-rearranged NSCLC [overall response rate (ORR): 18%-37%; median progression-free survival (PFS): 2.3-5.5 months)] (Drilon et al., 2016; Gautschi et al., 2017)], is clearly inferior compared with responses and survival outcomes seen with selective tyrosine kinase inhibitors (TKIs) and other driver oncogenes, such as *EGFR* (ORR 56%-85% and median PFS 9.2-13.7 months) (Castellanos et al., 2017), *ALK* (ORR 60%-95% and median PFS 8–11 months) (Caccese et al., 2016), and *ROS1* (ORR 65%-85% and median PFS 9.1-19.3 months) rearrangements (Facchinetti et al., 2017). In the case of RXDX-105, tumors arising from fusions between *KIF5B* and *RET* (0/20) did not respond to the drug, whereas patients with non-KIF5B-RET fusions showed an ORR of 67% (6/9) in a phase I/1B clinical trial (Drilon et al., 2019a), further raising concerns about the ability of MKIs to inhibit only select RET fusions. In addition, MKIs, i.e. cabozantinib, are often associated with a high rate of drug-related toxicities, leading to dose reductions in up to 70% of patients (Drilon et al., 2016). Selective inhibition of RET has shown more promise in lung cancer. In a phase 1 trial of pralsetinib (BLU-667), the ORR was 58% in *RET* fusion-positive NSCLC (Gainor et al., 2019). Similarly, selpercatinib has shown an ORR of 64% in *RET* fusion-positive NSCLC patients, who had previously been treated with RET therapy and an ORR of 85% in patients, who had not received previous RET therapy (Drilon et al., 2019b, 2020; Wirth et al., 2019).

There is no comprehensive study of the efficacy of any RET inhibitor in preclinical lung cancer models with *RET* fusions (Drilon et al., 2019b, 2020; Gainor et al., 2019). Indeed, the efficacy of cabozantinib, selpercatinib and pralsetinib has only been reported in one patient-derived lung cancer cell line, i.e. LC-2/ad (Li et al., 2017), which is likely to be due to the paucity of patient-derived disease models. In our study here, we developed four new patient-derived lung cancer models with different RET fusions and examined the efficacy of cabozantinib in these models. We further analyzed TCGA data sets to examine the signaling pathways that are activated in lung cancers with *RET* rearrangements with multiple upstream fusion partners. We found that cabozantinib effectively inhibits growth of *RET*-rearranged cell lines harboring oncogenic *CCDC6-RET*, *TRIM33-RET* or *KIF5B-RET* fusions, and is effective *in vivo* in patient-derived xenografts. Importantly, cabozantinib was effective in a KIF5B-RET model that did not respond to RXDX-105. We further found that the MYC pathway is activated by *RET* fusions, and that expression of MYC protein is decreased in response to treatment with cabozantinib, suggesting that a combination therapy of RET and novel MYC inhibitors may improve patient outcomes.

## RESULTS

### Generation and characterization of novel *RET-*rearranged cell lines

**Fig. 1.**
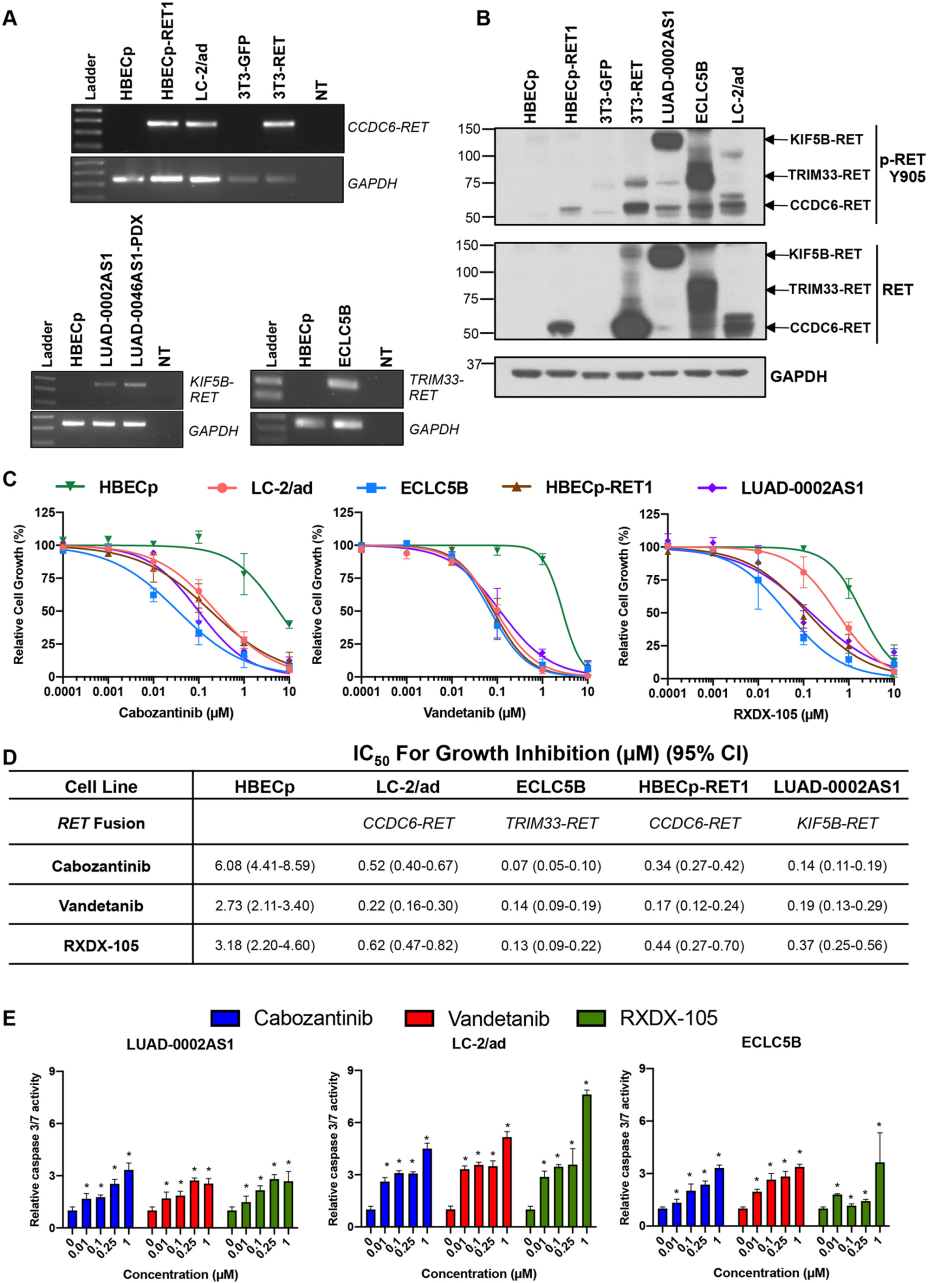
**RET fusion-positive cell lines are sensitive to multi-kinase RET inhibitors.** (A) *RET* gene expression was confirmed by using RT-PCR in LC-2/ad and novel isogenic cell lines, and analyzed by agarose gel electrophoresis. (B) Levels of RET and phosphorylated RET (p-RET Y905) were analyzed by western blotting of whole-cell extracts as indicated. (C) Cells were starved of serum for 24 h, treated with the indicated concentrations of cabozantinib, vandetanib, or RXDX-105 for 96 h, and then cell viability (cell growth in %) was determined using alamarBlue viability dye. (D) Data were analyzed by non-linear regression using GraphPad Prism; IC_50_ values are shown with the 95% confidence interval (CI) in brackets. Data represent the mean±s.e. of three (RXDX-105 and vandetanib) or five (cabozantinib) independent experiments, in which each condition was assayed in triplicate determinations. (E) Caspase 3/7 activity. Different cell lines were treated with the indicated concentrations of cabozantinib, vandetanib or RXDX-105 for 48 h upon which caspase 3/7 enzymatic activity was determined in each case. Results represent the mean±s.d. of two independent experiments in which each condition was assayed in triplicate determinations. *P<0.05, two-tailed *t*-test. NT, no template.

To perform preclinical investigation of RET inhibitors, we compiled a collection of *RET*-driven cell lines. This collection included the previously reported LC-2/ad cell line that harbors a *CCDC6-RET* fusion (Li et al., 2017) (xxxxx) as well as two novel patient-derived cell lines generated by us, i.e. ECLC5B harboring *TRIM33-RET* and PDX-derived lung adenocarcinoma cells harboring *KIF5B-RET* (LUAD-0002AS1). The patient demographic details and genomic profiling of the novel models by the MSK-IMPACT platform is given in Table S1 and Table S2, respectively. Additionally, we have established isogenic cell lines by overexpressing the *CCDC6-RET* fusion gene in 3T3 murine fibroblasts (NIH 3T3) and immortalized human broncho-epithelial cells (HBECp) yielding 3T3-RET and HBECp-RET1, respectively. We first examined expression of the respective *RET* fusion mRNA by PCR using primers that bind to 5′ partners and exon 12 of *RET*, respectively (Fig. 1A). Protein expression and phosphorylation were confirmed by western blotting (Fig. 1B). We used antibodies against total RET and against RET fusion protein phosphorylated at tyrosine residue Y905 (p-RET Y905), and found multiple bands in lysates extracted from some cell lines, possibly representing different isoforms, i.e. in the case of the patient-derived and PDX-derived cell lines, or post-translational modifications of RET (Fig. 1B).

### Cabozantinib inhibits growth and induces apoptosis in cell lines harboring RET fusions

We next evaluated the effect of cabozantinib on growth of RET fusion-positive or control cells. As comparison, we used two other MKIs, i.e. vandetanib and RXDX-105, shown to inhibit RET (Gautschi et al., 2017; Drilon et al., 2019a). While the non-tumor HBECp cell line displayed only marginal sensitivity to these compounds, growth of RET-driven cell lines was reduced at low nanomolar concentrations of the RET inhibitors (Fig. 1C,D). ECLC5B cells (TRIM33-RET) were the most responsive to cabozantinib and RXDX-105. Growth of all RET fusion-driven cell lines was inhibited in response to vandetanib − with similar IC_50s_ values. We further assessed the ability of these compounds to induce apoptosis by analyzing activation of caspase 3/7. Treatment of cells with cabozantinib for 48 h resulted in a small but significant dose-dependent activation of caspase 3/7 in the three cell lines tested (Fig. 1E). Similarly, vandetanib and RXDX-105 stimulated caspase 3/7 in a dose-dependent manner (Fig. 1E).

### Signaling pathways activated by RET activation

**Fig. 2.**
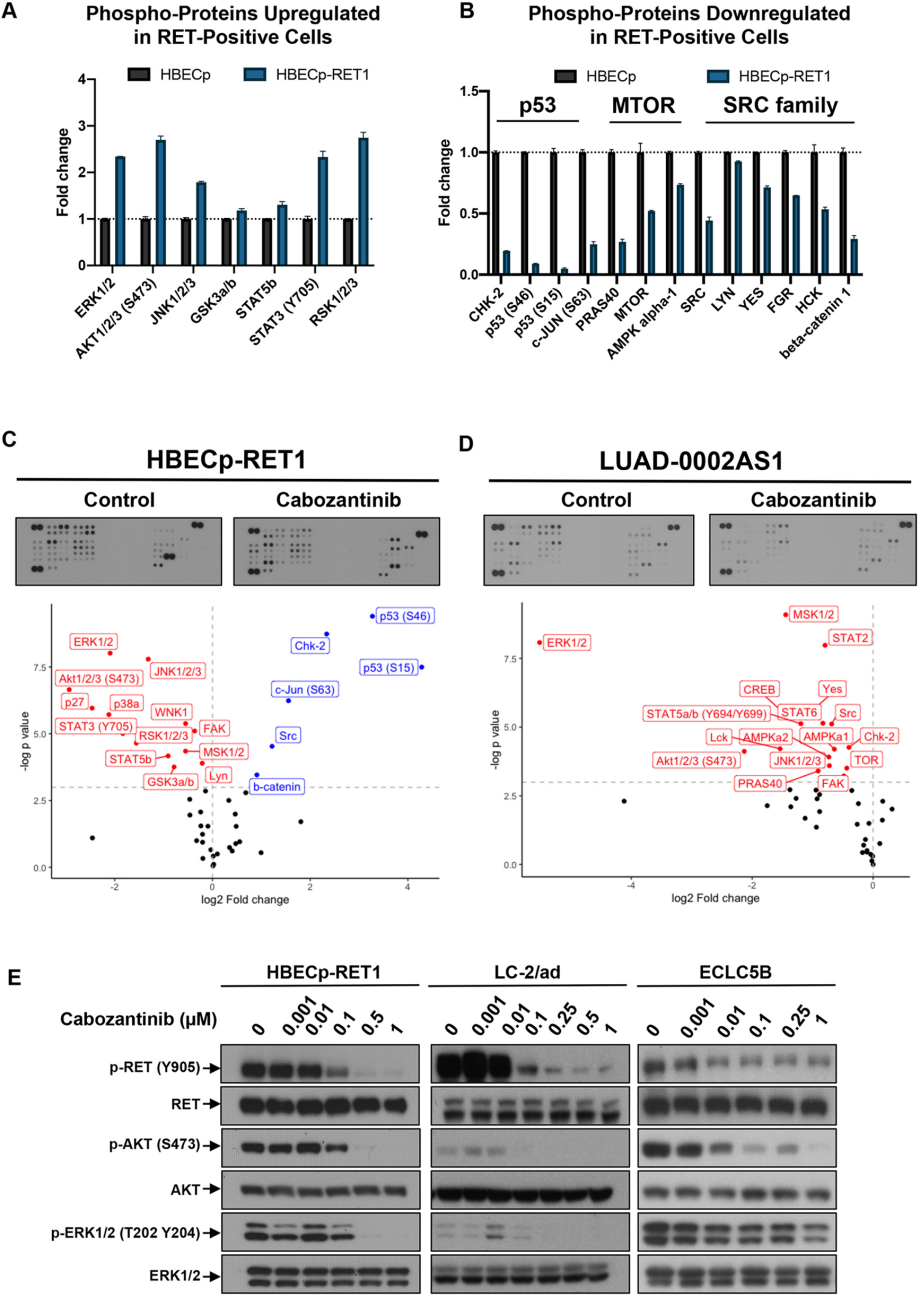
**RET fusion proteins regulate distinct pathways.** (A,B) Proteomic profiling of phospho-kinases was conducted in duplicates, in untreated HBECp and HBECp-RET1 cells. The relative change in phosphorylation between the two cell lines is shown as protein phosphorylation upregulated (A) or downregulated (B) following CCDC6-RET expression, as quantified from two technical replicates. Only proteins with significant change in phosphorylation are shown (*P*<0.05). (C,D) HBECp-RET1 (C) or LUAD-0002AS1 (D) cells were treated with 0.25 µM cabozantinib for 1.5 h, followed by proteomic profiling of phospho-kinases in their cell extracts. Phosphorylation of proteins was quantified from two technical replicates and expressed relative to control (DMSO-treated) cells in the graphs. Two-tailed *t*-test was used to calculate the *P*-value. Protein values shown in red indicate loss of phosphorylation, those in blue indicate increase in phosphorylation. (E) HBECp-RET1, LC-2/ad and ECLC5B cells were treated with the indicated concentrations of cabozantinib for 1.5 h followed by western blot analysis of their cell extracts for levels of phorphorylated and total kinases as indicated. Three independent experiments were conducted. Representative immunoblots of two experiments are shown.

To identify cell signaling pathways activated by RET, we analyzed the phosphorylation profile of 43 kinases involved in RTK-induced signaling within HBECp and RET-transformed, HBECp-RET1 cells. Expression of CCDC6-RET caused significant increases in phosphorylation of ERK1/2, RSK1/2/3, AKT1/2/3, JNK1/2/3, STAT3, STAT5B and GSK3a/b (Fig. 2A). Three main signaling axes were identified for the downregulated phospho-proteins, namely, CHK2-TP53, PRAS40-MTOR and SRC family kinases (Fig. 2B). Additionally, levels of phospho-JUN and total beta-catenin were significantly decreased in cells expressing the CCDC6-RET fusion protein (Fig. 2A,B).

We further treated HBECp-RET1 cells with cabozantinib to evaluate its effect on the phosphorylation state of 43 kinases. Remarkably, cabozantinib significantly decreased the phosphorylation of all proteins that were significantly phosphorylated by RET expression, restored phosphorylation of the CHK2-p53 axis, SRC and JUN, and expression of beta-catenin (Fig. 2C). Phosphorylation levels of PRAS40 and MTOR were also upregulated after treatment with cabozantinib, although these changes did not reach statistical significance. These findings were largely confirmed in LUAD-0002AS1 cells that harbor a *KIF5B-RET* fusion, and in which treatment with cabozantinib resulted in decreased phosphorylation of ERK1/2, AKT1/2/3, JNK1/2/3, STAT2, STAT5 and STAT6 (Fig. 2D). Interestingly, phosphorylation of SRC family kinases (SRC, YES, LCK) was decreased in LUAD-0002AS1 cells after cabozantinib treatment, consistent with a previous report of prominent Src activation in a *Drosophila* model of *KIF5B-RET* fusion (Das and Cagan, 2017). Of note, phosphorylation of PRAS40, MTOR (TOR), AMPKa1, AMPKa2 and CHK2 was also decreased after treatment with cabozantinib, suggesting additional differences beyond those previously reported (Das and Cagan, 2017) exist between different *RET* fusions (Fig. 2D). We confirmed that phosphorylation of AKT and ERK is inhibited upon treatment with cabozantinib by western blot analysis of HBECp-RET1, LC-2/ad and ECLC5B whole-cell extracts (Fig. 2E).

### *RET*-driven lung tumors demonstrate MYC transcriptional signatures that are inhibited upon treatment with cabozantinib

**Fig. 3.**
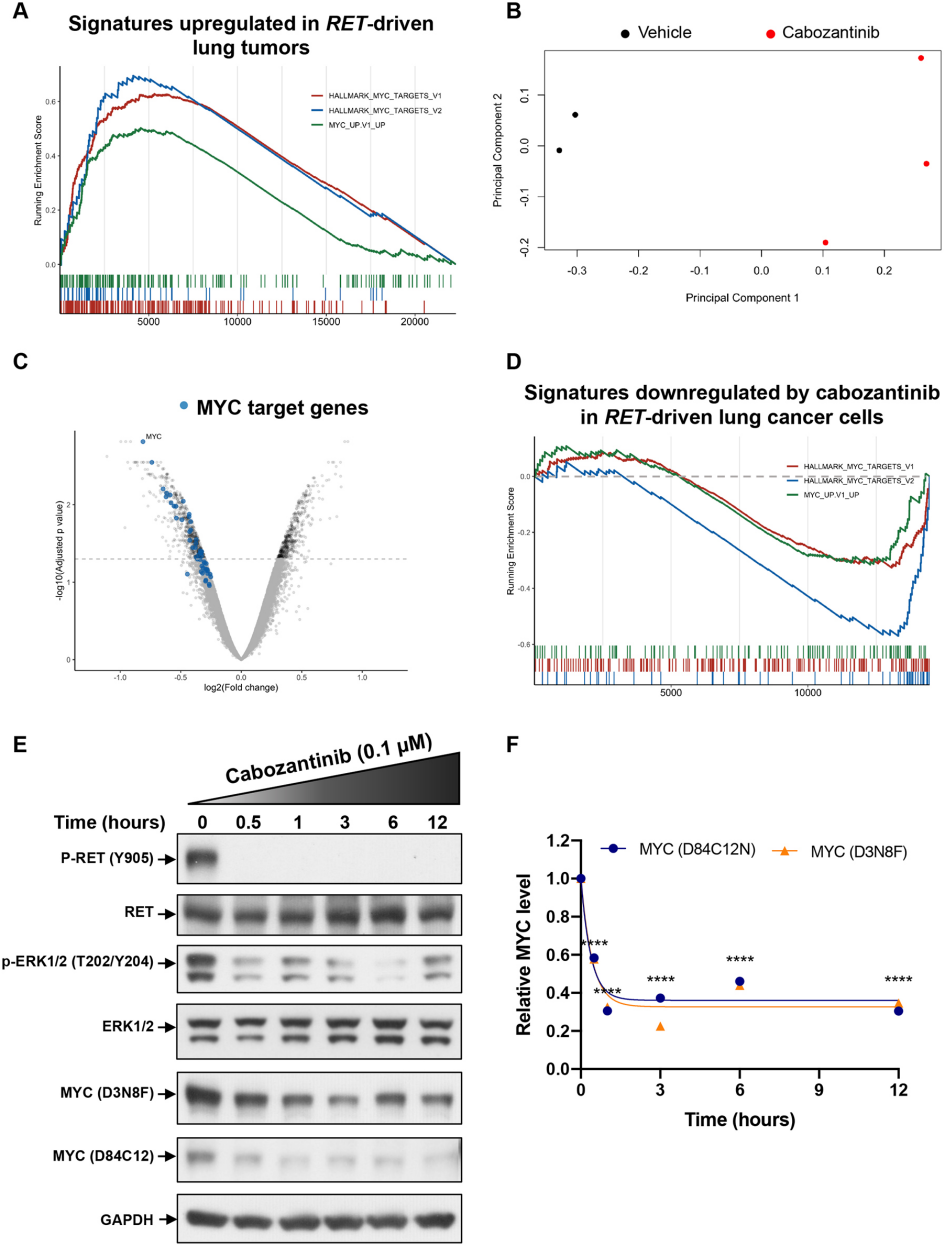
**MYC signaling and expression is regulated by RET in lung cancers with *RET* alterations.** (A) GSEA of the transcriptome of TCGA lung cancer tumors compared with that of normal lung samples. ECLC5B cells were treated with 0.1 µM cabozantinib for 3 h and the RNA isolated for transcriptomic profiling. (B-D) Principal component analysis (B), mRNA expression (C) and GSEA analysis (D) are shown. MYC target genes (leading edge genes from HALLMARK MYC TARGETS V1 and V2 combined) are indicated by blue dots. (E,F) Western blot analysis of MYC levels in ECLC5B cells following treatment with 0.1 µM cabozantinib for the indicated times. Representative immunoblots are shown (E). Immunoblots generated using two independent anti-MYC antibodies were scanned (*n*=2) and quantitated, and the half-life for MYC was estimated using GraphPad Prism (F). ****P<0.0001, two-tailed *t*-test.

To identify transcriptomic changes associated with *RET* expression, we analyzed RNA sequencing data obtained by The Cancer Genome Atlas (TCGA) for lung adenocarcinoma. We identified three cases (two *RET* fusions, one activating point mutation) and compared their transcriptomes to healthy lung tissues. Exploratory data analysis indicated that *RET*-driven tumors segregated from normal lung tissue as expected (data not shown). The output of differential gene expression analysis was ranked and followed by gene set enrichment analysis using Hallmark and Oncogenic signature gene sets from the Molecular Signature Database. We identified significant activation of MYC-associated transcriptional signatures (HALLMARK_MYC_TARGETS_V1, HALLMARK_MYC-TARGETS_V2, MYC_UP.V1_UP) (Fig. 3A). The full results of differential gene expression analysis and gene set enrichment analysis (GSEA) for RET-driven LUAD cells are summarized in Tables S3 and S4, respectively.

To investigate the effect of cabozantinib on RET fusion-associated gene expression, we treated the ECLC5B cell line, which is the most sensitive to treatment with cabozantinib, for 3 h and performed gene expression analysis. Fig. 3B demonstrates principal component analysis indicating different grouping of untreated and cabozantinib-treated samples and Fig. S1 demonstrates a summary heatmap of the differential expression analysis. Remarkably, the *MYC* oncogene was one of the five most-downregulated genes, accompanied by many associated transcriptional targets (Fig. 3C). GSEA demonstrated that RET-associated MYC transcriptional signatures were significantly downregulated by cabozantinib (HALLMARK_MYC_TARGETS_V1, HALLMARK_MYC_TARGETS_V2, MYC_UP.V1_UP) (Fig. 3D). Furthermore, western blotting analysis using two anti-MYC polyclonal antibodies targeting independent epitopes revealed a rapid and significant decline in MYC protein levels following cabozantinib treatment (0.1 µM), plateauing at the 1 h time point (Fig. 3E,F). The rapid decrease in MYC level (half-time <30 min) suggests that expression of MYC requires continuous input from activated RET. The full results of differential expression analysis and GSEA for ECLC5B cells treated with cabozantinib are summarized in Tables S5 and S6, respectively.

We further performed overlapping analysis of the two expression datasets to determine genes that are significantly upregulated (adjusted *P*-value <0.05) in RET-driven LUADs and downregulated after cabozantinib treatment (Table S7). We identified multiple genes with oncogenic properties such as *POLD2* (DNA polymerase delta 2, participating in DNA replication), *MCM5* (minichromosome maintenance complex component 5, involved in the initiation of DNA replication), *NGF* (nerve growth factor), TUBA4A (tubulin alpha 4a, participating in cell division) and *ETV4*, an oncogenic transcription factor previously demonstrated as crucial for RET signaling (Lu et al., 2009).

### Efficacy of cabozantinib administration on growth of RET inhibitor treatment-naive preclinical models with RET fusions

**Fig. 4.**
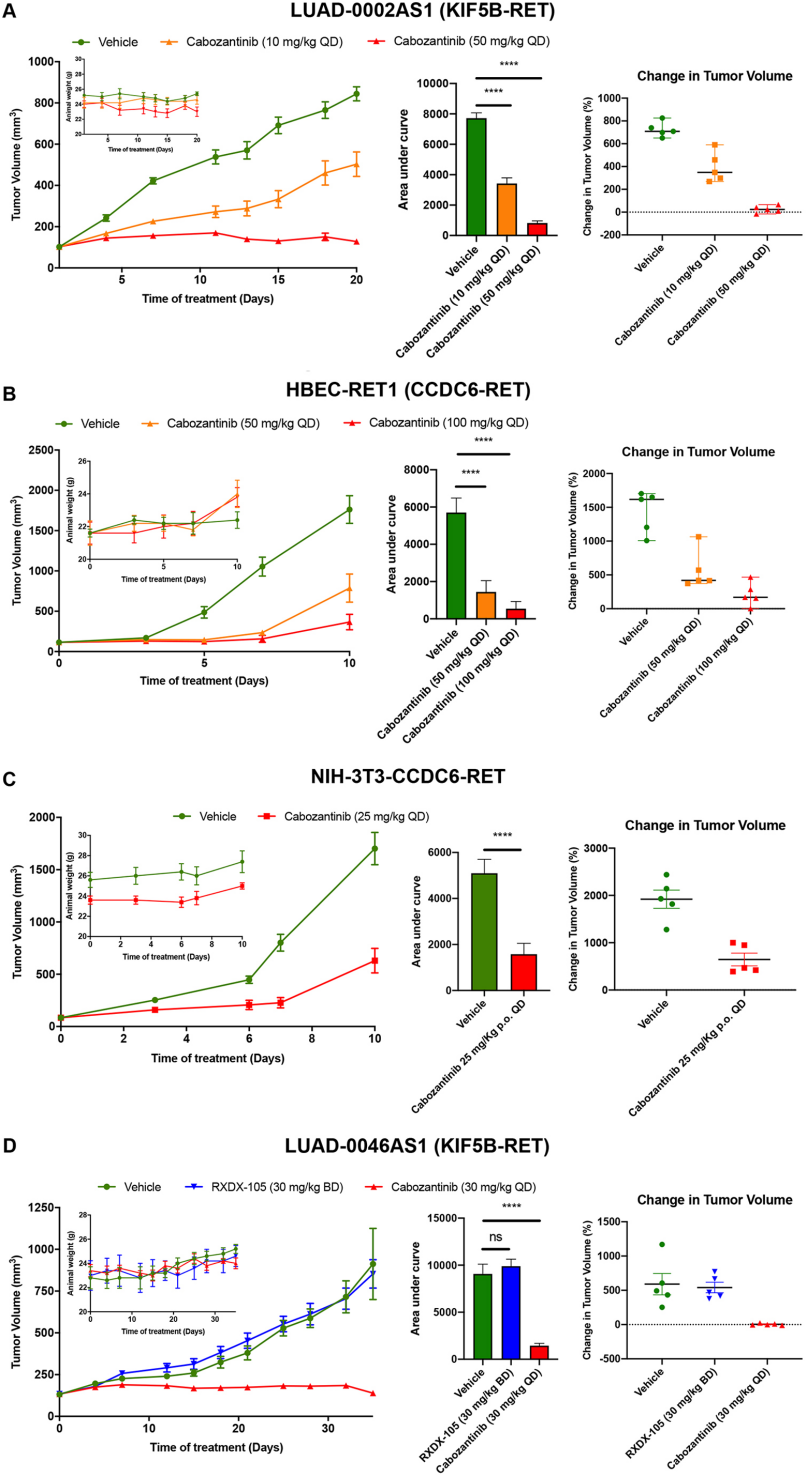
**Cabozantinib inhibits the growth of cell-line and patient-derived xenografts and overcomes RXDX-105 resistance in a PDX model.** (A-D) PDX tumors (A,D) or cells (B,C) were implanted into the subcutaneous flank of NSG mice. Treatment with cabozantinib and RXDX-105 was initiated once tumors had reached ∼100 mm^3^. Average tumor volumes (left panels), animal weight (left panels, insets), area under the curve analysis (middle panel) and the change in tumor volume (in %) of individual tumors (right panels) are shown. Per group, five mice were analyzed. Graphs and data analysis were conducted using GraphPad Prism. ns, not significant, ****P<0.0001, two-tailed *t*-test.

To determine how effective cabozantinib is *in vivo*, we treated mice bearing PDX or engineered cell line xenograft tumors obtained from our RET fusion-driven models with cabozantinib and assessed growth over time. Animals were treated with doses in the range of 10-100 mg/kg QD. These doses were based on previous *in vivo* studies in mice showing that 30 mg/kg cabozantinib caused >50% inhibition of phosphorylation of the RET fusion protein at tyrosine residue Y905, and complete inhibition at 60 mg/kg (Paratala et al., 2018). Fig. 4A, shows the tumor volume (left panel), area under the curve analysis to compare response over the entire time of treatment (middle panel) and the size of individual tumors at the end of the study (right panel) of each tumor. Treatment of mice bearing LUAD-0002AS1 PDX tumors (KIF5B-RET) resulted in a significant reduction of tumor growth following administration of 10 mg/kg QD (*P*=0.001, 40% difference between vehicle and treatment) or 50 mg/kg QD (*P*<0.0001, 85% difference between vehicle and treatment). Cabozantinib was compared to the vehicle-treated group at the end of the study (Fig. 4A, left panel). In both treatment groups, tumor growth was significantly reduced in response to cabozantinib administration (Fig. 4A, middle panel), with all tumors showing reduction in growth (Fig. 4A, right panel). Treatment with 50 mg/kg cabozantinib resulted in complete inhibition of growth. Administration of cabozantinib did not affect animal weight (Fig. 4A, inset). We next determined the efficacy of cabozantinib on the growth of tumors arising from isogenic HBEC and NIH-3T3 cell lines expressing a CCDC6-RET fusion. The tumor volume (left panel), area under the curve analysis to compare response over the entire time of treatment (middle panel) and the size or individual tumors at the end of the study (right panel) of each tumor are shown in Fig. 4B and C. As observed with the KIF5B-RET-positive PDX models, cabozantinib caused significant reductions in tumor growth at the doses tested in the two models (Fig. 4B,C). Cabozantinib treatment blocked tumor growth completely for the first 10 and 15 days, respectively, for the NIH-3T3-CCDC6/RET and HBECp-RET1 models. However, NIH-3T3-CCDC6/RET and HBECp-RET1 xenograft tumors started to regrow towards the end of the treatment periods (Fig. 4B,C, left panels). We did not observe any statistically significant reduction in animal weight in any of the cabozantinib treatment groups (Fig. 4B,C, left panel, inset).

### Efficacy of cabozantinib on the tumor growth of an RXDX-105-resistant PDX model

To expand these efficacy studies, we examined the effect of cabozantinib on the growth of PDX tumors (LUAD-0046AS1) that had been derived from a patient who did not respond to RXDX-105. Tumor-bearing mice were treated with vehicle, cabozantinib (30 mg/kg QD) or RXDX-105 (30 mg/kg BID). As expected, treatment with RXDX-105 did not significantly affect growth of LUAD-0046AS1 PDX tumors (KIF5B-RET) (vehicle: 912.3±213.1 mm^3^; RXDX-105: 853.2±84.7 mm^3^) (Fig. 4D). However, treatment with cabozantinib blocked growth of LUAD-0046AS1 PDX tumors completely (starting volume: 133.8±2.3 mm^3^; end volume: 139±9.6 mm^3^). This dosage of RXDX-105 was previously shown to reduce growth of RET fusion-driven cell lines xenograft tumors (Li et al., 2017). All tumors in the cabozantinib treatment group responded to the drug (Fig. 4D, right panel). There was no significant reduction in animal weight in any of the treatment arms (Fig. 4D, left panel, inset).

## DISCUSSION

Owing to a lack of preclinical models of lung cancer with *RET* rearrangements there have been very few studies examining the efficacy of RET inhibitors and signaling pathways exploited by RET fusions to drive tumorigenesis prior to clinical trials. Here, we generated four novel preclinical patient-derived models of RET-rearranged lung cancers and used these models to examine the efficacy of the RET MKI cabozantinib. In addition, we sought to identify the biochemical pathways that are required to drive RET fusion-dependent growth in these models.

We found that cabozantinib was effective *in vitro* and *in vivo*, in models with three different 5′ *RET* fusion partners, including two models harboring a *KIF5B-RET* fusion that has previously been shown to respond poorly to RXDX-105 (Drilon et al., 2019a). Cabozantinib inhibited growth of several cell lines and animal models carrying *RET* fusions. Of note, cabozantinib was effective in a *KIF5B-RET* PDX model derived from a patient tissue that did not respond to RXDX-105. Our profiling of RET-associated phospho-proteome signaling in isogenic cell lines that harbor a CCDC6-RET fusion, demonstrated activation of ERK, AKT and STAT, as well as suppression of CHK2 and P53. Treatment with cabozantinib reversed these changes. Although most of our findings were confirmed in a PDX-derived cell line harboring a *KIF5B-RET* fusion, a peculiar discrepancy occurred in the response of SRC family kinases. Here, inhibition of SRC phosphorylation was only seen in cells harboring KIF5B-RET fusion; cells with CCDC6-RET fusion showed increased SRC phosphorylation. This finding is congruent with the previously demonstrated ability of *KIF5B-RET* fusion to induce activation, i.e. phosphorylation, of SRC and EGFR/FGFR (Das and Cagan, 2017). Accordingly, a phase 1/2 clinical study of the multi-targeted RET and SRC kinase inhibitor TPX-0046 (NCT04161391), is ongoing for TKI-naive and TKI-pretreated patients with RET-altered lung, thyroid and other cancers (Drilon et al., 2019c).

To gain insight into how RET fusions may activate growth, we used transcriptomic profiling of RET-driven lung adenocarcinomas, which highlighted an enhanced *MYC* signature in these tumors. Correspondingly, cabozantinib treatment of a *RET* fusion-positive cell line led to downregulation of the *MYC* oncogene, suppression of the *MYC* signature and decreased levels of MYC protein. These results indicate that RET inhibitors can regulate *MYC* transcription. Although it is possible that MYC protein degradation is also enhanced, our study did not specifically address this. Alterations in MYC signaling are hallmarks of many human cancers, such as small cell lung cancer and Burkitt lymphoma (Dalla-Favera et al., 1982; Kim et al., 2016), and alterations in the MYC pathway have been observed in ∼30% of cancers with diverse histologies (Schaub et al., 2018). Although the connection between the MYC pathway and oncogenesis is recognized, this is the first report of a potential role for MYC in RET-induced tumorigenesis. Silencing of *MYC* in multiple tumor models by using small interfering (si)RNAs leads to tumor regression (Han et al., 2019; Jain et al., 2002; Shachaf and Felsher, 2005), suggesting that MYC may be exploited for therapy. Although direct pharmacological inhibition of MYC remains a challenge, recent studies have shed a promising light on targeting this potent transcription factor (Han et al., 2019). Our finding that MYC expression requires continued signaling from RET opens a potentially new avenue for the combinatorial targeting of RET-fusion-driven lung cancers, with RET and new MYC inhibitors to improve response rates.

As mutational activation of downstream signaling mediators is a demonstrated mechanism of resistance to RTK inhibitors, it is important to map RET-associated signaling pathways to properly investigate and predict mechanisms of resistance, and to identify opportunities for therapeutic strategies to overcome this resistance. We used four novel patient-derived preclinical models to begin to map RET fusion-associated signaling. These models will be invaluable in future testing of new therapy, and in the development of models of RET-inhibitor resistance *in vitro* and *in vivo* to profile drug-resistance mechanisms.

## MATERIALS AND METHODS

### Compounds and cell lines

The human lung adenocarcinoma cell line LC-2/ad (Cat. No. 94072247) was obtained from Riken Cell Bank (Japan). The HBECp-RET1 cell line was generated by expressing CCDC6-RET and a dominant-negative p53 (C-terminal region of wild-type p53) (Sasai et al., 2011) in human bronchial epithelial cells immortalized by CDK4 and TERT overexpression (HBEC3-KT) cells (Sato et al., 2006) and was described previously (Li et al., 2017). All cell lines were tested every 6 months for mycoplasma and *RET* fusion verified by PCR each time a new frozen vial of stock was thawed. Novel cell lines and PDXs were verified by MSK-IMPACT NGS testing. All primary antibodies used in the present study were obtained from Cell Signaling Technology (Danvers, MA, USA) and were validated by the manufacturer. Antibodies against phosphorylated RET (Y905; #3221), RET (#3220), phosphorylated ERK (T202/Y204; #9101), ERK (#4695), phosphorylated AKT (S473; #4060), AKT (#4691), GAPDH (#5174), and MYC (D3N8F, #13987, targets the central region and D84C12, #5605, targets the amino terminal region) were all used at 1:1000 dilution. All cell culture growth media (except for keratinocyte serum-free medium), antibiotics and phosphate-buffered saline (PBS) were prepared by the MSK Media Preparation Core Facility. Fetal bovine serum (FBS), proteome profiling arrays and secondary antibodies conjugated to horseradish peroxidase (HRP) were procured from R&D Systems (Minneapolis, MN, USA). RXDX-105 (agerafenib, CEP32496), cabozantinib, lenvatinib, vandetanib, and ponatinib were purchased from Selleckchem (Houston, TX, USA). Promega's ApoOne Homogenous Caspase 3/7 activity assay kit, alamarBlue viability dye, keratinocyte serum-free medium (KSM), tissue culture plastic wares, NuPAGE gels, blotting buffers and all western blotting reagents were obtained from ThermoFisher Scientific (Waltham, MA, USA). Protease and phosphatase inhibitor cocktails, RIPA lysis buffer (10×) and all other chemicals not listed above were purchased from EMD-Millipore Sigma (St Louis, MO, USA). All oligonucleotides used for PCR assays were obtained from Integrated DNA technologies (Coralville, IA, USA).

### Growth and propagation of cell lines

All cell lines were grown in a humidified incubator under 5% CO_2_ at 37°C. Cells were maintained in 75-cm^2^ flasks and, when approaching 75% confluence, subcultured using 0.25% trypsin/1 mM EDTA solution. ECLC5B and LUAD-0002AS1 cells were maintained in DMEM/F12 supplemented with 10% (vol/vol) FBS and 1% (vol/vol) antibiotic solution. LC-2/ad and HBECp-RET1 cells were maintained in RPMI-1640 medium supplemented with 10% (vol/vol) FBS and 1% (vol/vol) antibiotic solution. HBEC3-KT cells were maintained in keratinocyte serum-free medium supplemented with 50 µg/ml bovine pituitary extract (BPE) and 5 ng/ml recombinant epidermal growth factor (EGF). NIH-3T3 and NIH-3T3-CCDC6/RET cell lines were grown in DMEM supplemented with 10% FBS.

### Generation of cell lines and patient-derived xenografts

Tissue samples were collected under an MSK institutional IRB-approved biospecimen collection protocol and all patients provided informed consent for collection and use of tissues. Animals were monitored daily and cared for in accordance with guidelines approved by the Memorial Sloan Kettering Cancer Center Institutional Animal Care and Use Committee and Research Animal Resource Center. To obtain pleura effusion a thoracentesis was performed on a patient with acquired resistance to cabozantinib and samples were collected in a sterile container in which heparin was added (10 USP units/ml fluid). Epithelial cells were isolated by differential centrifugation on Ficoll gradient. To obtain a single cell suspension, cells were treated with trypsin, neutralized with DMEM/F12 medium supplemented with 10% FBS, filtered through a 40 µM cell strainer, pelleted and re-suspended in DMEM/F12 medium with 10% FBS. The cabozantinib-resistant cell line was considered established after 20 continuous passages and named ECLC5. Given the rarity of RET-inhibitor sensitive cell lines with confirmed RET fusions, we sought to establish a subline with enhanced sensitivity to cabozantinib. A previous study has shown that TKI sensitivity can be restored to drug-resistant tumor cells in culture by withdrawing the selection pressure (cabozantinib in our case) (Chmielecki et al., 2011). We, therefore, cultured ECLC5 long term in the absence of cabozantinib, to allow any drug-responsive subclones of cells to gain a proliferative advantage. This cabozantinib-sensitive subpopulation was named ECLC5B and was used for experiments in this study. Patient-derived xenograft models were generated by implanting biopsied lung tumor samples [minced and then mixed with 50% (vol/vol) matrigel] in the subcutaneous flank of female NOD/SCID gamma (NSG) mice (Jackson Laboratory, Bar Harbor, ME). PDX tumors were transplanted for at least 3 serial passages before the model was considered established and used for efficacy studies. The LUAD-0046AS1 PDX model was created from tissue obtained from a patient who was treated with RXDX-105 but did not respond. The LUAD-0002AS1 PDX model was derived from a patient who never received any RET therapy. The LUAD-0002AS1 cell line was created from the 4th passage of PDX tissue. Briefly, fresh tumors were cut into small pieces and then digested in a cocktail of tumor dissociation enzymes obtained from Miltenyl Biotec (130-095-929) in 5 ml serum-free DME:F12 medium for 1 h, 37°C, with vortexing every 5-10 min, according to manufacturer's instructions. Digested samples were resuspended in 45 ml complete growth medium to inactivate the dissociation enzymes and then cells pelleted by centrifugation. Finally, cells were plated in complete growth medium and allowed to propagate over multiple generations, trypsinized when necessary to subculture and eventually only single cells remained. The cell line was considered established after 20 continuous passages.

### Genomic characterization

Cell lines and PDX were profiled by the MSK-IMPACT (Integrated Mutation Profiling of Actionable Cancer Targets) platform, which is a large panel sequencing (NGS) assay, that was used here to detect mutations and copy-number alterations involving up to 468 cancer-associated genes (Cheng et al., 2015). As the corresponding patient-matched normal DNA was unavailable, single nucleotide variants (SNVs) representing known COSMIC somatic mutations or truncating mutations in tumor suppressor genes and copy number variants (CNVs) were tabulated.

### Efficacy studies in xenograft models

For efficacy studies, xenograft tumor samples were cleaned and then minced, mixed with matrigel and implanted into a subcutaneous flank of female NSG mice to generate xenografts. Tumor-bearing animals were randomized to groups of five when tumors reached approximately 100 mm^3^ volume so that averages of tumor volume within and between groups were similar. Treatment was initiated with vehicle, cabozantinib (at 10, 25, 30, 50 or 100 mg/kg QD) or RXDX-105 (30 mg/kg BID). Cabozantinib was reconstituted in a vehicle consisting of 30% polypropylene glycol, 5% Tween-80 and 65% D5W (dextrose 5% water) and RXDX-105 was resuspended in 15% captisol. Both compounds were administered by oral gavage. Tumor size and body weight were measured twice weekly, and tumor volume was calculated using the formula: length×width2×0.52.

### Cell growth, drug treatments, viability and caspase 3/7 activity assays

Cells were plated directly into chemicals at a density of 6000 (to assess viability) or 20,000 (to assess caspase 3/7 activity) cells per well in white, clear bottom 96-well plates. Viability was assayed after 96 h of treatment using alamarBlue viability dye, fluorescence (Ex: 530 nm, Em: 570 nm) was measured using a Spectramax M2 microplate reader (Molecular Devices, Sunny Vale, CA). Data were analyzed by non-linear regression using GraphPad Prism v8.0 to obtain IC_50_ values. To assess induction of apoptosis, caspase 3/7 enzymatic assay was performed 48 h after treatments using the ApoOne homogeneous caspase 3/7 activity kit according to manufacturer's instruction. Each condition was assayed in triplicate determinations in at least two experiments. For western blot analyses, cells were plated at a density of 10^6^ cells per well in 6-well plates and used 4 days later. Cells were deprived of serum for 24 h prior to treatment, and all treatments were conducted in serum-free medium.

### Isolation of RNA, cDNA synthesis and RT-PCR

For detection of the *RET* fusion transcript in cell lines and PDX tissues, total RNA were extracted using a Qiagen RNA mini kit and cDNAs were synthesized using SuperScript IV VILO (ThermoFisher Scientific) according to the manufacturer's instructions. The following primers were used for RT-PCR: *GAPDH* Fw 5′-GGC GCT GAG TAC GTC GTG GAG TCC-3′, *GAPDH* Rv 5′-AAA GTT GTC ATG GAT GAC CTT GG-3′, *CCDC6* exon 1 Fw 5′-GCA TTG TCA TCT CGC CGT TCC G-3′, *KIF5B* exon 15 Fw 5′-GCA ACT TTA GCG AGT ATA GAT-3′, *TRIM33* exon 14 Fw 5′-AGC AAG AAC CTG GGA CTG AAG ATG-3′, *RET* exon 12 Rv 5′-TGC TCT GCC TTT CAG ATG GAA GG-3′.

### Preparation of whole-cell extracts and western blotting

Cells were lysed in 350 µl RIPA lysis buffer that was supplemented with 1 mM sodium orthovanadate, 1 mM DTT (dithiothreitol) and protease and phosphatase inhibitor cocktails according to the manufacturer's instructions. Lysates were denatured in 2×Laemmli sample buffer at 55°C for 5 min, resolved on 4-12% NuPAGE reducing gels and transferred onto PVDF (polyvinylidene fluoride) membranes. Membranes were blocked in 3% BSA in TBST buffer (Tris-buffered saline with 0.1% Tween-20) for 1 h at room temperature and probed with primary antibodies overnight. Bound antibodies were detected with peroxidase-labeled goat anti-mouse or goat anti-rabbit IgG and developed with ECL western blotting detection reagents. Images were captured on X-ray film. All experiments were repeated at least twice.

### Phospho-kinases – proteomic profiling array

We used a Phospho-Kinase Array Kit containing duplicate validated controls and capture antibodies that simultaneously detect the relative phosphorylation state of 43 human kinases (R&D Systems), according to the manufacturer's protocol. A total of 5×10^6^ cells were plated in 10 cm dishes and then deprived of serum for 24 h. In brief, the array membranes were blocked, incubated with 300 µg cell lysates per array overnight at 4°C. The next day arrays were washed three times, incubated with biotinylated antibodies for 2 h at room temperature, washed three times, incubated with streptavidin-HRP for 30 min at room temperature, washed again three times and developed with ECL western blotting detection reagents. The kinase spots were visualized with X-ray films (Ewen-Parker X-ray). The average pixel densities of duplicate spots were determined using the ImageJ software (http://imagej.nih.gov/ij/).

### TCGA RNAseq expression analysis

Gene expression data from The Cancer Genome Atlas were retrieved from the public functional genomics data repository Gene Expression Omnibus (accession GSE62944) (Rahman et al., 2015). Data were normalized and analyzed using DESeq2 package in the R programming environment. Gene set enrichment analysis (GSEA) was conducted and visualized using clusterProfileR R package. Additionally, ggplot2 R package was used for data visualization.

### Gene expression microarray analysis

ECLC5B cells were plated at a density of 500,000 cells per well in 6-well plates and then treated 24 h later with 0.1 µM cabozantinib or 0.1% DMSO in triplicates. After 3 h, cells were washed with ice-cold PBS and total RNA was extracted using RNeasy mini kit (Qiagen, Germantown, MD). All microarray hybridization and scanning steps were performed by the MSK Integrated Genomics Operations Core Laboratory. Briefly, total RNA was converted to double-stranded DNA using oligo d(T) primers and reverse transcriptase before *in vitro* transcription with biotinylated UTP and CTP. The resulting biotinylated RNA was then fragmented and hybridized for 16 h at 45°C to the Affymetrix oligonucleotide Human HG-U133A Genechip (Santa Clara, CA), containing 22,215 probe sets representing ∼18,500 transcripts and 14,500 genes. The resulting data were analyzed using R programming environment. First, data was Robust Multichip Average (RMA)-normalized and unsupervised clustering profiling was performed using principal component analysis. One outlier sample was identified in the untreated group and removed from further analysis. Linear regression analysis was performed using limma R package to detect differentially expressed genes. GSEA was conducted and visualized using clusterProfileR R package.

### Statistical analysis

Student's *t*-test was used to compare caspase activity or protein phosphorylation. For animal studies, area under the curve analysis was used to compare the average tumor volume between groups. Briefly, area under the curve values and standard errors were computed as an estimation of the surface area between baseline values (mean value of the tumor volumes at the beginning of the treatment) and growth curves for vehicle and each treatment conditions. Treatment response was compared to the vehicle group using multiple Student's *t*-tests. All data were plotted and analyzed using GraphPad Prism v8 software. *P*<0.05 was considered significant.
